# Synthesis of Disaccharides Containing 6-Deoxy-α-L-talose as Potential Heparan Sulfate Mimetics

**DOI:** 10.3390/molecules17089790

**Published:** 2012-08-15

**Authors:** Jon K. Fairweather, Ligong Liu, Tomislav Karoli, Vito Ferro

**Affiliations:** Drug Design Group, Progen Pharmaceuticals Ltd, Brisbane QLD 4076, Australia

**Keywords:** disaccharides, heparan sulfate mimetics, fibroblast growth factors

## Abstract

A 6-deoxy-α-L-talopyranoside acceptor was readily prepared from methyl α-L-rhamnopyranoside and glycosylated with thiogalactoside donors using NIS/TfOH as the promoter to give good yields of the desired α-linked disaccharide (69–90%). Glycosylation with a 2-azido-2-deoxy-D-glucosyl trichloroacetimidate donor was not completely stereoselective (α:β = 6:1), but the desired α-linked disaccharide could be isolated in good overall yield (60%) following conversion into its corresponding tribenzoate derivative. The disaccharides were designed to mimic the heparan sulfate (HS) disaccharide GlcN(*2S*,*6S*)-IdoA(*2S*). However, the intermediates readily derived from these disaccharides were not stable to the sulfonation/deacylation conditions required for their conversion into the target HS mimetics.

## 1. Introduction

The fibroblast growth factors FGF-1 and FGF-2 are heparan sulfate (HS)-binding proteins that play key roles in tumor angiogenesis, a critically important process in tumor growth and development [1,2]. They promote angiogenesis by binding with HS and their receptors (FGFRs) to form a ternary HS:FGF:FGFR complex which leads to receptor dimerization/activation and subsequent initiation of cell signaling [3]. Inhibiting angiogenesis by blocking ternary complex formation with HS mimetics is thus a promising strategy for the development of anticancer drugs [4,5,6] without the side effects sometimes associated with other antiangiogenic therapies [7].

**Figure 1:**
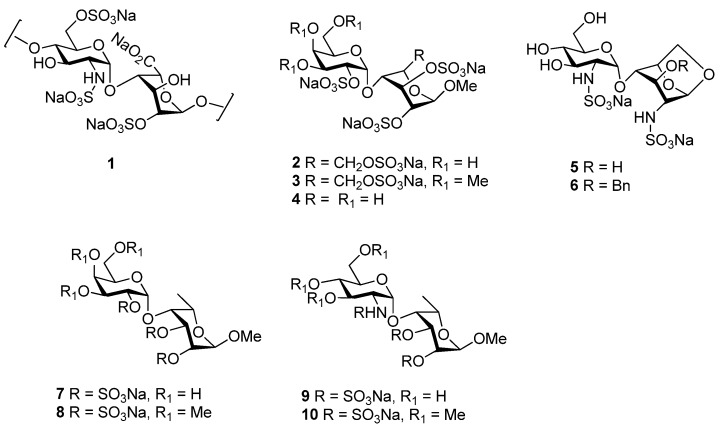
Structures of the GlcN(*2S*,*6S*)-IdoA(*2S*) disaccharide sequence **1**, which represents a minimal consensus sequence for FGF:HS binding [20], conformationally flexible mimetics **2**–**4** [18], conformationally locked mimetics **5** and **6** [19], and proposed disaccharides of intermediate flexibility **7**–**10**.

A number of studies have described the synthesis of specific HS or HS-like oligosaccharides to interact with FGF-1 or FGF-2 [8,9,10,11] in order to obtain information about structural requirements for HS-FGF binding and activation. Despite much recent progress [12,13,14], the synthesis of native HS oligosaccharides remains a difficult and labour-intensive exercise and has thus lead to interest in less synthetically challenging oligosaccharide mimetics as FGF antagonists [10,15,16,17]. As part of a program aimed at the development of angiogenesis inhibitors, we recently described [18,19] the synthesis of simple disaccharides such as **2**–**6** which mimic the HS disaccharide GlcN(*2S*,*6S*)-IdoA(*2S*) (**1**, Figure 1), postulated from X-ray crystallographic analyses as a minimal HS consensus sequence for FGF binding [20]. The compounds were designed to maintain the α-(1→4) linkage between the two monosaccharide units and the spatial orientation of the two key sulfo groups [GlcN(*2S*) and IdoA(*2S*)]. The conformationally flexible disaccharides **2**–**4** were designed to mimic this known property [21,22] of IdoA residues. Disaccharides **5** and **6**, on the other hand, were designed to investigate the other extreme: a locked ^1^*C*_4_ conformation. Molecular docking calculations indicated that the predicted locations of disaccharide sulfo groups in the binding site of FGF-1 and FGF-2 were consistent with the positions observed for co-crystallized heparin-derived oligosaccharides. These studies suggest that it may be possible to mimic HS oligosaccharides with simpler structures.

In order to extend the above investigations we herein describe the design and synthesis of simple disaccharides with intermediate conformational flexibility compared with **2**–**6**, which once sulfated, could mimic disaccharide **1**.

## 2. Results and Discussion

**Figure 2:**
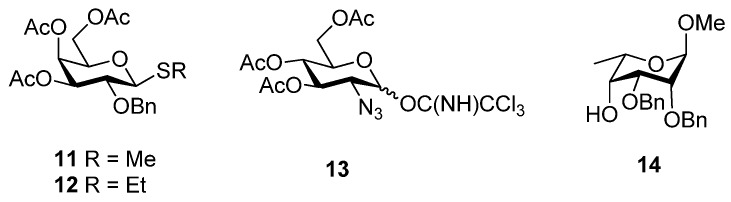
Glycosyl donors (**11**–**13**) and 6-deoxy-L-taloside acceptor (**14**).

The previously synthesized disaccharides **2**–**6** were designed to mimic the essential features of **1**, in particular the α-(1→4) linkage between the two monosaccharide units and the spatial orientation of the two key sulfo groups [GlcN(*2S*) and IdoA(*2S*)], with the assumption that *N*-sulfo groups could be interchanged with *O*-sulfo groups and vice versa. The disaccharides were prepared by the glycosylation of suitable monosaccharide acceptors (as IdoA mimics) with non-participating C2-protected glycosyl donors such as D-thiogalactosides **11** and **12** or the 2-azido-glucosyl imidate **13** (Figure 2), to favour the stereoselective formation of the desired α-(1→4) linkage. Disaccharides **2**–**4** are composed of a 2-*O*-sulfated D-Gal α(1→4)-linked to a polysulfated β-D-glucoside or xyloside. Polysulfation of β-D-glucosides or xylosides confers conformational flexibility upon this monosaccharide residue [23,24], confirmed by ^1^H-NMR spectroscopy, thus mimicking the conformational flexibility of IdoA. Disaccharides **5**–**6** on the other hand are composed of a D-glucosamine-*N*-sulfate α(1→4)-linked to a 1,6-anhydro-2-amino glucose. The latter is locked in the ^1^*C*_4_ conformation, mimicking the conformation of IdoA as found in some crystal structures of heparin oligosaccahrides bound to FGF [25,26].

**Scheme 1:**
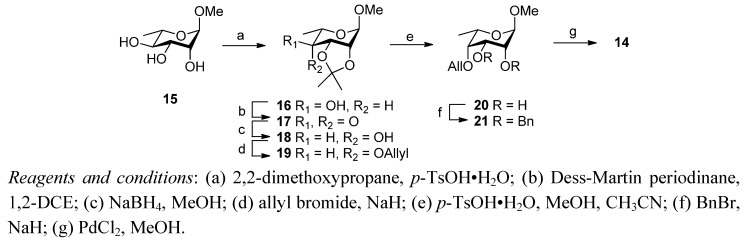
Synthesis of 6-deoxy-L-taloside acceptor **14**.

In order to extend the above studies, it was decided to investigate IdoA mimics with intermediate degrees of conformational flexibility. The 6-deoxy-L-taloside **14** was thus selected as a potential glycosyl acceptor because, like the majority of the L-sugars, it was expected to adopt the ^1^*C*_4_ conformation in solution but not be strictly held in this conformation like **5** and **6**. It was anticipated that the use of **14** would, after deprotection and sulfonation, lead to target disaccharides such as **7**–**10**. In addition to the desired 2-*O*-sulfate, an additional sulfate at *O*-3 could provide additional electrostatic interactions with the target proteins. Acceptor **14** was thus prepared in a straightforward manner from methyl α-L-rhamnopyranoside **15** [27], as outlined in Scheme 1. Triol **15** was treated with 2,2-dimethoxypropane and toluenesulfonic acid as catalyst to give the isopropylidene **16** which was subsequently oxidised with Dess-Martin periodinane to the ketone **17** in good yield (70%, 2 steps). Stereoselective reduction with sodium borohydride in methanol gave exclusively the 6-deoxy-α-L-taloside **18** which was converted into the diol **20** in moderate yield (54%, 3 steps) via allylation at C4 followed by toluenesulfonic acid catalysed methanolysis of the isopropylidene group. Diol **20** was then benzylated (NaH/benzyl bromide, 87%) and de-*O*-allylated with PdCl_2_ in methanol at reflux to afford the alcohol **14** in excellent yield (95%), ready for use in the glycosylation studies.

**Scheme 2:**
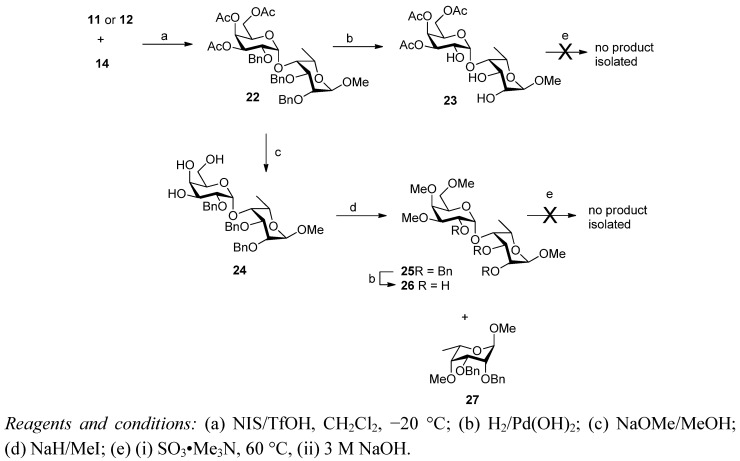
Assembly of D-Gal-L-Tal disaccharide and attempted conversion into sulfated derivatives.

Glycosylation of acceptor **14** with methyl thiogalactoside donor **11** in dichloromethane at −20 °C with NIS/TfOH as the promoter was very rapid and gave the disaccharide **22** in high yield (90%) following purification by flash chromatography (Scheme 2). Analysis of the ^1^H-NMR spectrum of **22** confirmed the presence of the newly formed α-glycosidic linkage (doublet at 5.77 ppm, *J*_1,2_ = 3.6 Hz), and that the L-taloside ring remained in the desired ^1^*C*_4_ conformation (^3^*J* = 2.4–3.2 Hz). Interestingly the use of the homologous ethyl donor **12** resulted in a less clean reaction and only gave **22** in a still acceptable 69% isolated yield after flash chromatography.

Attention was then turned towards conversion of **22** into the sulfated target disaccharides **7** and **8**. Unfortunately, the compounds in this series proved to be unusually unstable to the standard transformations [18,19] used to successfully prepare disaccharides **2**–**6** (Scheme 2). Hydrogenolytic debenzylation of disaccharide **22** was hampered by apparent poisoning of the palladium catalyst by trace sulfur-containing impurities from the glycosylation step. However, by replacing the catalyst four times during reaction, and in the presence of glacial acetic acid, triol **23** was obtained in low yield (33%), but good purity after flash chromatography. Subsequent attempted sulfonation with concomitant deacetylation of **23** (SO_3_.Me_3_N followed by aqueous 3 M NaOH) gave rise to complex mixtures from which no pure product could be isolated by the chromatographic procedures previously used [18,19] (size exclusion chromatography on Bio-Gel P-2). It is known that sulfonation of carbohydrate polyols with sulfur trioxide-amine complexes can induce cleavage of acid labile groups and glycosidic linkages [28]. Evidently disaccharide **23** is not stable to these harsh conditions. In attempts to prepare the trimethyl derivative, both the Zemplén deacetylation/methylation and hydrogenolysis steps were low yielding (29–46% and 55%, respectively). The former caused significant degradation and in one case resulted in the isolation of the monosaccharide **27** as the dominant product (71%). Cleavage of glycosidic bonds under basic conditions in the presence of atmospheric oxygen is known [29,30], and could account for the degradation seen here, but it is unclear why these disaccharides are so sensitive compared with the earlier series. Attempted sulfonation of the small amounts of available **26** produced also gave rise to complex mixtures from which the desired products could not be isolated.

**Scheme 3:**
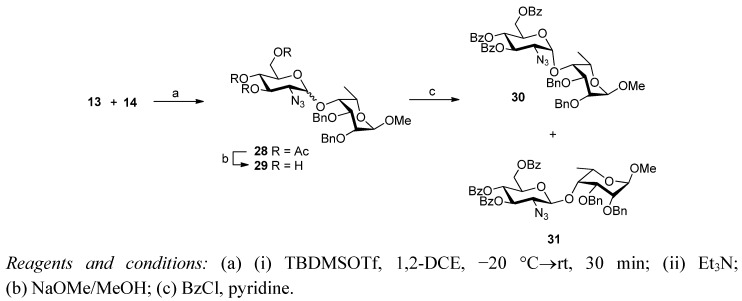
Synthesis of D-Glc-L-Tal disaccharides.

Attention was then turned to the alternative disaccharide series using imidate **13** as the glycosyl donor (Scheme 3). Following literature precedent [31], TBDMSOTf was selected as the promoter for the glycosylation of **14** with donor **13**. The reaction proceeded rapidly in 1,2-dichloroethane at −20 °C (10 min, then→rt over 20 min), however, it was not completely stereoselective and resulted in an inseparable mixture of α and β anomers **28** (α:β = 6:1). The crude mixture was therefore deacetylated under Zemplén conditions (NaOMe in MeOH) and the crude triol **29** then benzoylated with benzoyl chloride in pyridine. The resultant mixture of benzoates was separable by careful column chromatography from which the desired α-linked disaccharide **30** was isolated in 60% overall yield along with the β-anomer **31** (10%).

**Scheme 4:**
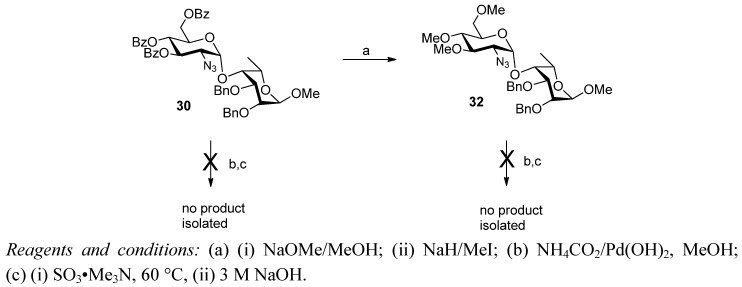
Attempted conversion into sulfated derivatives.

We were not able to transform disaccharide **30** into the desired sulfated products **9** or **10** (Scheme 4). Disaccharide **30** was subjected to catalytic transfer hydrogenation (Pearlman’s catalyst/ammonium formate) to presumably give the crude amine. However, attempted sulfonation only gave a complex mixture of products and ^1^H-NMR analysis indicated some loss of benzoates. The mixture was subjected to standard benzoylation conditions (excess benzoyl chloride/pyridine) but this did not result in simplification of the mixture and no pure products could be recovered. Compound **30** was subjected to the Zemplén and methylation procedures to give the trimethyl derivative **32** in moderate yield (69% over 2 steps). However, when this compound was subjected to the same azide reduction/sulfonation procedure as above, once again a complex mixture resulted from which no identifiable products were isolated.

## 3. Experimental

### General

^1^H-NMR spectra were recorded at 400 MHz for ^1^H, 100 MHz for ^13^C in deuteriochloroform (CDCl_3_) with residual CHCl_3_ (^1^H, δ 7.26) employed as internal standard, at ambient temperatures (298 K) unless specified otherwise. Where appropriate, analysis of ^1^H-NMR spectra was aided by gCOSY experiments. Flash chromatography was performed on Merck silica gel (40–63 μm) under a positive pressure with the specified eluants. All solvents used were of analytical grade. The progress of the reactions was monitored by TLC using commercially prepared Merck silica gel 60 F_254_ aluminium-backed plates. Compounds were visualized by charring with 5% sulfuric acid in MeOH and/or by visualization under ultraviolet light. The term ‘workup’ refers to dilution with water, extraction into an organic solvent, sequential washing of the organic extract with aq. 1 M HCl (where appropriate), saturated aq. NaHCO_3_ and brine, followed by drying over anhydrous MgSO_4_, filtration and evaporation of the solvent by means of a rotary evaporator at reduced pressure and where appropriate, extensive drying of the residue at <1 mmHg.

*Attempted sulfonation*. The polyol was dissolved in anhydrous DMF (0.04 M) and sulfur trioxide pyridine complex (2 eq. per hydroxyl) or sulfur trioxide trimethylamine complex (3 eq. per hydroxyl) was added. The mixture was stirred at 60 °C under a nitrogen (6–16 h), cooled (0 °C), treated with MeOH (2 mL) and then made basic to pH ≥ 9 by addition of 3 M NaOH solution. The mixture was filtered and evaporated to dryness and the residue was purified by size exclusion chromatography (Bio-Gel P-2, 5 × 100 cm, 2.8 mL/min, 0.1 M NH_4_HCO_3_, 2.8 min per vial). The fractions were analyzed for carbohydrate content by TLC (charring) or the 1,9-dimethylmethylene blue test [32] and for purity by CE [33]. 

*Attempted sulfonation/deacylation*. The polyol was sulfonated according to the general procedure for sulfonation, however, the residue obtained from evaporation of basified (pH = 9) crude mixture was redissolved in 3 M NaOH (0.16 M) and stirred at rt (o/n) before purification.

*Ethyl 3,4,6-tri-O-acetyl-2-O-benzyl-1-thio-β-D-galactopyranoside* **12**. The title compound was prepared from ethyl 1-thio-β-D-galactopyranoside in an analogous fashion to the methyl thiogalactoside **11** according to the procedure of Pozsgay [34]. Flash chromatography (hexanes/EtOAc 6:1→2:1) gave **12** as a colourless oil (R*_f_* = 0.20, hexanes/EtOAc 4:1). ^1^H-NMR: δ 7.35-7.23 (m, 5H, Ph), 5.37 (dd, 1H, *J*_3,4_ = 3.2, *J*_4,5_ = 1.2, H4), 4.98 (dd, 1H, *J*_2,3_ = 9.6, H3), 4.83, 4.57 (ABq, 2H, *J*_A,B_ = 10.8, C*H*_2_Ph), 4.52 (d, 1H, *J*_1,2_ = 9.6, H1), 4.13 (dd, 1H, *J*_6a,6b_ = 11.2, *J*_5,6a_ = 7, H6a), 4.05 (dd, 1H, *J*_5,6b_ = 6.4, H6b), 3.84 (ddd, 1H, H5), 3.62 (dd, 1H, H2), 2.82-2.68 (m, 2H, SCH_2_), 2.09 (s, 3H, Ac), 2.00 (s, 3H, Ac), 1.90 (s, 3H, Ac), 1.30 (t, 3H, *J* = 7.6, CH_3_).

*Methyl 6-deoxy-2,3-O-isopropylidene-α-L-lyxo-hexopyran-4-uloside* **17**. (A) *p*-TsOH.H_2_O (100 mg) was added to a mixture of methyl α-L-rhamnopyranoside (**15**) [27] (1.45 g, 8.1 mmol) in 2,2-dimethoxypropane (10 mL) and the combined mixture stirred (rt, 20 min). Et_3_N (100 μL) was added to neutralise the reaction mixture and the solvent was evaporated. The residue was dissolved (EtOAc) and subjected to workup yielding, presumably, the acetal **16** [35] as a colourless oil. This was used for the next step without further purification. (B) Dess-Martin periodinane (3.80 g, 8.9 mmol) was added to the crude alcohol **16** [from (A) above] in 1,2-dichloroethane and the combined mixture heated (70 °C, 1 h). The mixture was cooled (rt) and then diluted (CHCl_3_) prior to workup including pre-treatment with Na_2_S_2_O_3_ (2 M). The residue was subjected to flash chromatography (EtOAc/hexanes 1:9→3:7) to give the ketone **17** [36] as a pale yellow oil (1.24 g, 70%, 2 steps). This was used for the next step without further characterisation.

*Methyl 4-O-allyl-6-deoxy-α-L-talopyranoside* **20**. (A) NaBH_4_ (200 mg, 5.0 mmol) was added portion-wise to a stirred solution of the ketone **17** (1.24 g, 5.7 mmol) in MeOH (60 mL). The mixture was treated with AcOH (10% aq.) dropwise to destroy the excess reducing agent and the solvent evaporated. The residue was subjected to workup (EtOAc) yielding the alcohol **18** as a pale yellow oil. This was used in the next reaction without further purification. (B) The alcohol **18** [from (A) above] in DMF (2 mL) was added dropwise to a stirred suspension of pre-washed (hexane) NaH (560 mg of 50% oil suspension, 11.4 mmol) in DMF (15 mL) and the combined mixture stirred (0 °C→rt, 30 min). The mixture was then cooled (0 °C) and allyl bromide (735 μL, 8.5 mmol) was introduced and stirring continued (0 °C→rt, o/n). The mixture was cooled (0 °C), MeOH (3 mL) was added and stirring continued (5 min) prior to evaporation of the solvent. The residual oil was subjected to workup (EtOAc) to yield the acetal **19** as a pale yellow oil (1.12 g). This was used for the next reaction without further purification. (C) A mixture of the acetal **19** [from (B) above] and *p*-TsOH.H_2_O (100 mg) in MeOH (20 mL) and MeCN (20 mL) was heated under reflux (1 h). The mixture was cooled (rt) and Et_3_N (100 μL) was added prior to evaporation of the solvent. The residue was subjected to workup (EtOAc) and flash chromatography (EtOAc/hexanes 1:4→2:3) to yield the diol **20** as a colourless oil (668 mg, 54%, 3 steps). ^1^H-NMR: δ 1.26 (d, 3H, *J*_5,6_ 6.4 Hz; H6), 3.33 (s, 3H, OMe), 3.46–3.48 (m, 1H, H2), 3.62 (br s, 1H, H4), 3.75 (dd, 1H, *J*_2,3_ = *J*_3,4_ = 3.2 Hz, H3), 3.79-3.85 (m, 1H H5), 4.12–4.26 (m, 2H, OCH_2_), 4.71 (s, 1H, H1), 5.15–5.27 (m, 2H, CH=C*H*_2_), 5.84–5.93 (m, 1H, C*H*=CH_2_); ^13^C-NMR (CDCl_3_, 100 MHz): δ 17.1, 55.2, 65.9, 66.7, 70.9, 75.6, 81.3, 102.2, 118.0, 134.3.

*Methyl 2,3-di-O-benzyl-6-deoxy-α-L-talopyranoside* **14**. (A) The diol **20** (110 mg, 0.50 mmol) in DMF (1 mL) was added dropwise to a stirred suspension of pre-washed (hexane) NaH (500 mg of 50% oil suspension, 10 mmol) in DMF (5 mL) and the combined mixture stirred (0 °C→rt, 30 min). The mixture was then cooled (0 °C) and benzyl bromide (250 μL), 2.0 mmol) was introduced and stirring continued (0 °C→rt, o/n). The mixture was cooled (0 °C) and MeOH (3 mL) was added with continued stirring (5 min) prior to evaporation of the solvent. The residual oil was subjected to rapid silica filtration (10–40% EtOAc/hexanes) to yield, presumably, the dibenzyl ether **21** as a pale yellow oil (174 mg, 87%). This was used for the next reaction without further characterisation or purification. (B) A mixture of the allyl ether **21** (170 mg, 0.43 mmol) and PdCl_2_ (20 mg) in MeOH (15 mL) was heated under reflux (1 h). The solvent was evaporated and the residue subjected to flash chromatography (EtOAc/hexanes 1:9→3:7) to yield the alcohol **14** as a pale yellow oil (146 mg, 95%). ^1^H-NMR: δ 1.30 (s, 3 H, H6), 3.29 (s, 3 H, OMe), 3.65 (dd, 1 H, *J*_2,3_ = *J*_3,4_ = 3.2 Hz, H3), 3.69–3.75 (m, 3 H, H2, H4, H5), 4.51, 4.71 (ABq, *J*_A,B_ = 12.0 Hz, C*H*_2_Ph), 4.68, 4.79 (ABq, *J*_A,B_ = 11.9 Hz, C*H*_2_Ph), 4.72 (d, 1 H, *J*_1,2_ = 0.8 Hz, H1).

*Methyl 3,4,6-tri-O-acetyl-2-O-benzyl-α-D-galactopyranosyl-(1→4)-2,3-di-O-benzyl-6-deoxy-α-L-talo-pyranoside* **22**. *From donor **11**:* A mixture of the alcohol **14** (128 mg, 357 μmol), thioglycoside **11** (183 mg, 428 μmol) and freshly activated, powdered 3 Å mol sieves (500 mg) in dry CH_2_Cl_2_ (10 mL) was stirred at −30 °C for 20 min before adding NIS (125 mg, 556 μmol, 1.3 eq.) and TfOH (1 drop). Stirring was continued at −30 °C until the reaction was complete by TLC (~1.5 h) before Et_3_N (584 μL, 424 mg, 4.2 mmol, 7.5 eq.) was added. Evaporation onto silica gel and flash chromatography (EtOAc/hexanes 1:3→9:11) gave disaccharide **22** as a colourless film (238 mg, 90%; R*_f_* = 0.28, hexanes/EtOAc = 1:1). ^1^H-NMR: δ 7.1-7.4 (m, 15H, 3 × Ph), 5.77 (d, 1H, *J*_1,2_ = 3.6 Hz, H1^II^), 5.46 (dd, 1H, *J*_3,4_ = 3.4, *J*_4,5_ = 1.3 Hz, H4^II^), 5.41 (dd, 1H, *J*_2,3_ = 10.4 Hz, H3^II^), 4.83 (d, 1H, *J*_1,2_ = 2.4 Hz, H1^I^), 4.49-4.72 (m, 6H, PhC*H*_2_), 4.27 (ddd, 1H, H5^II^), 4.11 (dd, 1H, *J*_6a,6b_ = 11.4, *J*_5,6b_ = 7.0, H6b^II^), 4.04 (dd, 1H, *J*_5,6a_ = 6.4, H6a^II^), 3.95 (m, 1H, H5^I^), 3.94 (m, 1H, H4^I^), 3.83 (dd, 1H, H2^II^), 3.80 (dd, 1H, *J*_2,3~3,4_ = 3.2, H3^I^), 3.59 (dd, 1H, *J*_1,2+2,3_ = 2.8, H2^I^), 3.33 (s, 3H, OMe), 2.05 (s, 3H, Ac), 2.04 (s, 3H, Ac), 1.96 (s, 3H, Ac), 1.42 (d, 3H, *J*_5,6_ = 6.4, H6^I^); ^13^C NMR: δ 170.4, 170.0, 169.8, 138.3(2), 138.3(0), 138.1, 128.3, 128.1, 127.9, 127.8, 127.5, 127.4, 127.3(2), 127.2(8), 127.1, 99.0, 96.4, 77.3, 73.2, 72.3, 72.2, 72.1, 71.5, 71.2, 69.1, 68.4, 67.0, 66.3, 62.1, 55.0, 20.7, 20.6, 20.5, 17.0. *From donor **12**:* The alcohol **14** (95 mg, 265 μmol), thioglycoside **12** (117 mg, 265 μmol) and freshly activated, powdered 3 Å mol sieves (350 mg) were subjected to the above NIS glycosylation conditions using 78 mg (345 μmol, 1.3 eq.) of NIS. Flash chromatography (EtOAc/hexanes 1:4→1:1) gave the pure α-anomer **22** (134 mg, 69%).

*Methyl 3,4,6-tri-O-acetyl-α-D-galactopyranosyl-(1→4)-6-deoxy-α-L-talopyranoside* **23**. To a solution of **22** (120 mg, 163 μmol) in MeOH (5 mL) was added Pd/C (5%, 20 mg). The suspension was stirred (5 min) then filtered and additional Pd/C (5%, 20 mg) was added along with glacial acetic acid (20 μL) and the suspension was stirred overnight under hydrogen. The catalyst was replaced and the hydrogen was refilled with stirring overnight two more times before the suspension was filtered and subjected to flash chromatography (EtOAc/hexanes 3:2→7:3) to give the triol **23** (25 mg, 33%) as a colourless film. ^1^H-NMR (200 MHz, CDCl_3_): 5.44 (d, 1H, *J*_3,4_ = 3.1, H4^II^), 5.33 (d, 1H, *J*_1,2_ = 4.2, H1^II^), 5.09 (dd, 1H, *J*_2,3_ = 10.6, H3^II^), 4.73 (br s, 1H, H1^I^), 4.43 (t, 1H, *J*_5,6_ = 6.3, H5^II^), 4.11 (dd, 1H, H2^II^), 4.06 (app d, 2H), 3.8–4.0 (m, 3H), 3.65–3.71 (m, 1H), 3.35 (s, 3H, OMe), 3.3–3.5 (br s, 3H, OH), 2.12 (s, 3H, AcO), 2.03 (s, 3H, AcO), 2.02 (s, 3H, AcO), 1.32 (d, 3H, *J*_5,6_ = 6.6, H6^I^).

*Methyl 3,4,6-tri-O-methyl-2-O-benzyl-α-D-galactopyranosyl-(1→4)-2,3-di-O-benzyl-6-deoxy-α-L-talo-pyranoside* (**25**). A sliver of sodium was added to a solution of triacetate **22** (110 mg) in MeOH (10 mL) before stirring overnight. The solution was neutralised with resin (AG-50X8 H^+^), filtered and evaporated. DMF (10 mL), sodium hydride (30 mg, 60% in oil) and methyl iodide (50 μL) were added and the mixture was stirred overnight before it was quenched (ice), evaporated and subjected to flash chromatography (EtOAc/hexanes 3:7→3:2) to give **25** (45 mg, 46%) as a colourless film (R*_f_* = 0.08, hexanes/EtOAc = 65:35). ^1^H-NMR: δ 7.0–7.3 (m, 15H, 3 × Ph), 5.50 (br d, 1H, *J* = 3.1, H1^II^), 4.78 (d, 1H, *J* = 3.2, H1^I^), 4.4–4.7 (m, 6H, PhCH_2_), 3.9–4.0 (m, 2H), 3.82 (br t, 1H, *J* = 2.8), 3.81 (dd, 1H, *J* = 3.7, 10, H2^II^), 3.64–3.76 (m, 3H), 3.503 (s, 3H, OMe), 3.499 (s, 3H, OMe), 3.48–3.52 (m, 2H), 3.43 (dd, 1H, *J* = 6.2, 9.5), 3.34 (s, 3H, OMe), 3.29 (s, 3H, OMe), 1.37 (d, 3H, *J* = 6.7, H6^I^).

In a separate experiment, following the above deacetylation and benzylation procedures, the triacetate **22** (67 mg, 90.9 μmol) was converted to trimethyl ether **25** as a minor product (17 mg, 29%, R*_f_* = 0.08, hexanes/EtOAc = 65:35). The major fraction was the decomposed by-product methyl 2,3-di-*O*-benzyl-4-*O*-methyl-6-deoxy-α-L-talopyranoside **27** (colourless gum, 24 mg, 71%, R*_f_* = 0.32, hexanes/EtOAc 65:35). ^1^H-NMR: δ 7.42–7.24 (m, 10H, 2 × Ph), 4.86, 4.72 (ABq, 2H, *J*_A,B_ = 12.8, PhC*H*_2_), 4.76 (d, 1H, *J* = 1.6, H1), 4.53 (s, 2H, PhC*H*_2_), 3.85–3.80 (m, 1H), 3.68–3.64 (m, 2H), 3.66 (s, 3H, CH_3_O), 3.39 (m, 1H), 3.30 (s, 3H, CH_3_O), 1.33 (d, 3H, *J* = 6.4, CH_3_).

*Methyl 3,4,6-tri-O-methyl-α-D-galactopyranosyl-(1→4)-6-deoxy-α-L-talopyranoside* **26**. 5% Palladium on activated charcoal (10 mg) and acetic acid (200 μL) were added to a solution of tribenzyl ether **25** (40 mg, 61 μmol) in MeOH (10 mL). The reaction flask was evacuated and refilled with hydrogen three times before the mixture was stirred under hydrogen overnight. The mixture was then filtered, evaporated and subjected to flash chromatography (EtOAc) to yield of triol **26** (13 mg, 55%) as a colourless oil. ^1^H-NMR: δ 5.26 (d, 1H, *J* = 4.2, H1^II^), 4.76 (d, 1H, *J* = 1.4, H1^I^), 4.10-4.15 (m, 2H), 3.93 (dq, 1H, *J* = 0.7, 6.5, H5^I^), 3.82 (t, 1H, *J* = 3.4), 3.77–3.79 (m, 1H), 3.70 (dd, 1H, *J* = 1.4, 3.3, H2^I^), 3.55 (s, 3H, OMe), 3.52–3.58 (m, 1H), 3.50 (s, 3H, OMe), 3.42–3.47 (m, 2H), 3.38 (s, 3H, OMe), 3.36 (s, 3H, OMe), 1.32 (d, 3H, *J* = 6.8, H6^I^).

*Methyl 2-azido-3,4,6-tri-O-benzoyl-2-deoxy-α-D-glucopyranosyl-(1→4)-2,3-di-O-benzyl-6-deoxy-α-L-talopyranoside* **30**. (A) A mixture of the imidate [19] **13** (291 mg, 0.66 mmol) and alcohol **14** (157 mg, 0.44 mmol) in 1,2-DCE (5 mL) was stirred in the presence of activated mol. sieves (300 mg of 3 Å powder) under an atmosphere of argon (rt, 30 min) and then cooled (−20 °C) with continued stirring (10 min). TBDMSOTf (30 μL, 0.132 mmol) was introduced dropwise and the mixture was warmed (−20 °C→0 °C, 20 min). Et_3_N (100 μL) was introduced and the mixture was filtered and evaporated. The residue was subjected to workup (EtOAc) and flash chromatography (EtOAc/hexanes 1:9→2:3) to yield a fraction presumed to contain the disaccharide product **28** as a pale yellow oil (301 mg). ^1^H-NMR analysis indicated that a monosaccharide component was also present in the mixture. This residue was co-evaporated (2 × 10 mL CH_3_CN) and used in the next reaction without further purification or characterisation. (B) Na (a small piece) was added to a solution of the mixture from (A) (above) (0.44 mmol, max.) in MeOH (4 mL) and the combined mixture stirred (rt, 1 h). The mixture was evaporated and neutralised by the addition of Dowex 50X8 resin (H^+^ form), filtered and the filtrate evaporated. The residue was subjected to workup (EtOAc) and flash chromatography (EtOAc/hexanes 1:1→9:1) to yield a colourless oil (210 mg). This residue was co-evaporated (2 × 10 mL CH_3_CN) and used in the next reaction without further purification or characterisation. (C) BzCl (306 μL, 2.64 mmol) was added to a solution of the mixture from (A) (above) (**29**) (0.44 mmol, max.) and pyridine (2 mL) in 1,2-DCE (4 mL) and the combined mixture stirred (rt, o/n). The mixture was cooled (0 °C) and MeOH (2 mL) was introduced with continued stirring (0 °C→rt, 2 min) before evaporation and co-evaporation (toluene) of the solvent. The residue was subjected to workup (EtOAc) and flash chromatography (EtOAc/hexanes 1:9→3:7) to yield two compounds. Firstly, the α-linked disaccharide (**30**) was obtained as a colourless oil (217 mg, 60%, 3 steps). ^1^H-NMR: δ 1.48 (d, 3 H, *J*_5,6_ = 6.6 Hz, H6^I^), 3.31 (s, 3H, OCH_3_), 3.40 (dd, 1H, *J*_1,2_ = 3.7, *J*_2,3_ = 10.6 Hz, H2^II^), 3.64–3.65 (m, 1H, H2^I^), 3.73 (m, 1H, H3^I^), 3.88–3.95 (m, 1H, H5^I^), 4.05–4.07 (m, 1H, H4^I^), 4.35 (ddd, 1H, *J*_4,5_ = 10.2, *J*_5,6a_ = 3.3, *J*_5,6b_ = 5.8 Hz, H5^II^), 4.37, 4.61 (ABq, *J*_A,B_ = 11.6 Hz; C*H*_2_Ph), 4.50 (dd, 1H, *J*_6a,6b_ = 12.0 Hz, H6b^II^), 5.58 (dd, 1H, H6a^II^), 4.67, 4.77 (ABq, *J*_A,B_ = 12.7 Hz, C*H*_2_Ph), 4.82 (d, 1H, *J*_1,2_ = 1.6 Hz, H1^I^), 5.24 (dd, 1H, *J*_3,4_ = 9.5, *J*_4,5_ = 10.1 Hz, H4^II^), 5.99 (dd, 1H, *J*_2,3_ = 10.7, *J*_3,4_ = 9.3 Hz, H3^II^), 6.14–6.15 (d, 1H, *J*_1,2_ = 4.0 Hz, H1^II^), 7.20–7.54, 7.90–7.99 (2 m, 25H, 5 × Ph); ^13^C-NMR: δ 17.6, 55.2, 61.8, 63.7, 66.9, 68.3, 70.5, 70.6, 70.6, 74.5, 96.3, 99.9, 127.7, 128.2, 128.4, 128.5, 128.5, 128.6, 128.6, 129.0, 129.5, 129.8, 129.9, 130.0, 130.1, 133.3, 133.4, 133.6, 136.3, 138.8, 165.6, 165.7, 166.3.

Next, the β-linked disaccharide (**31**) was obtained as a colourless oil (36 mg, 10%, 3 steps). ^1^H-NMR: δ 1.46 (d, 3H, *J*_5,6_ = 6.6 Hz, H6^I^), 3.33 (s, 3H, OMe), 3.53 (t, 1H, *J*_1,2_ = *J*_2,3_ = 3.2 Hz, H2^I^), 3.70–3.75 (m, 1H, H2^II^), 3.85–3.93, 3.97–4.01 (2 m, 4H, H3^I^, H4^I^, H5^I^, H5^II^), 4.29 (dd, 1H, *J*_5,6a_ = 5.3, *J*_6a,6b_ = 12.2 Hz, H6a^II^); 4.44 (dd, 1H, *J*_5,6b_ = 3.3, H6b^II^), 4.54, 4.73 (ABq, *J*_A,B_ = 11.9 Hz, C*H*_2_Ph), 4.65 (d, 1H, *J*_1,2_ = 8.0 Hz, H1^II^), 4.72, 4.76 (ABq, *J*_A,B_ = 12.7 Hz, C*H*_2_Ph), 4.77 (d, 1H, *J*_1,2_ = 3.5 Hz, H1^I^), 5.44–5.51 (m, 2H, H3^II^, H4^II^), 7.20–7.51, 7.83–7.94 (2 m, 25H, 5 × Ph).

*Methyl 3,4,6-tri-O-methyl-2-azido-2-deoxy-α-D-glucopyranosyl-(1→4)-2,3-di-O-benzyl-6-deoxy-α-L-talopyranoside* **32**. Tribenzoate **30** (110 mg, 128 µmol) was subjected to the Zemplén and methylation procedures (as described above for the preparation of **25**) with MeI (82 mg, 582 µmol). Flash chromatography (hexanes→EtOAc/hexanes 1:4) gave the trimethyl derivative **32** (52 mg, 69%, 2 steps) as a colourless oil. ^1^H-NMR: δ 7.42–7.22 (m, 10H, 2 × Ph), 5.73 (d, 1H, *J* = 3.7, H1^II^), 4.84 (d, 1H, *J* = 2.0, H1^I^), 4.82, 4.77 (ABq, 2H, *J* = 12.6, C*H*_2_Ph), 4.58, 4.54 (ABq, 2H, *J* = 11.9, C*H*_2_Ph), 3.95–3.93 (m, 1H), 3.90 (dt, 1H, *J* = 2.0, 6.7, H5^I^), (s, 3H, OMe), 3.76–3.57 (m, 6H), 3.65, 3.55, 3.42, 3.31 (4 × s, 4 × 3H, OMe), 3.28–3.21 (m, 2H), 1.34 (d, 3H, *J* = 6.5, H6^I^).

## 4. Conclusions

In conclusion, the 6-deoxy-α-L-taloside acceptor **14** was readily prepared in seven steps from methyl α-L-rhamnopyranoside in good overall yield. Glycosylation of **14** with the thiogalactoside donors **11** or **12** with NIS/TfOH as the promoter gave good yields of the α-linked disaccharide **22**. Glycosylation with the thiomethyl donor **11** was preferred as it gave a cleaner reaction mixture from which the desired product was isolated in excellent yield. Glycosylation of **14** with the 2-azido-2-deoxy-glucosyl imidate **13** was not completely stereoselective (α:β = 6:1) and resulted in an inseparable mixture. However, deacetylation and conversion into the corresponding tribenzoates allowed for the isolation of the desired α-linked disaccharide **30** in good overall yield (60%). Unfortunately, the intermediates readily derived from **22** and **30** were not stable to the sulfonation/deacylation conditions required for their conversion into the target HS mimetics, resulting in complex mixtures from which no products could be isolated.

## Supplementary Materials

Supplementary materials can be accessed at: http://www.mdpi.com/1420-3049/17/8/9790/s1.
