# Supplementary material for: Synthesis of Disaccharides Containing 6-Deoxy-α-L-talose as Potential Heparan Sulfate Mimetics
Source: Molecules. 2012 Aug 15;17(8):9790–802. doi: 10.3390/molecules17089790 (PMC6268951; doi:10.3390/molecules17089790)
Supplement: Supplementary file 1 [file molecules-17-09790-s001.pdf]

Supplementary Data

Synthesis of Disaccharides Containing 6-deoxy-α-L-Talose as Potential Heparan Sulfate Mimetics

Jon K. Fairweather [1], Ligong Liu [1,2], Tomislav Karoli [1,2] and Vito Ferro [1,3]*

[1] Drug Design Group, Progen Pharmaceuticals Ltd, Brisbane QLD 4076, Australia
[2] Current address: The University of Queensland, Institute for Molecular Bioscience, Brisbane, QLD 4072, Australia
[3] Current address: The University of Queensland, School of Chemistry and Molecular Biosciences, Brisbane, QLD 4072, Australia

***** Author to whom correspondence should be addressed; E-Mail: v.ferro@uq.edu.au; Tel.: +61-7-3346-9598; Fax: +61-7-3365-4299.

Contents

^{1}H NMR (400 MHz) spectrum for compound **12** S3

^{1}H NMR (400 MHz) spectrum for compound **20** S4

^{13}C NMR (100 MHz) spectrum for compound **20** S5

^{1}H NMR (400 MHz) spectrum for compound **14** S6

^{1}H NMR (400 MHz) spectrum for compound **22** S7

^{13}C NMR (100 MHz) spectrum for compound **22** S8

^{1}H NMR (200 MHz) spectrum for compound **23** S9

^{1}H NMR (400 MHz) spectrum for compound **25** S10

^{1}H NMR (400 MHz) spectrum for compound **27** S11

^{1}H NMR (400 MHz) spectrum for compound **26** S12

^{1}H NMR (400 MHz) spectrum for compound **30** S13

^{13}C NMR (100 MHz) spectrum for compound **30** S14

^{1}H NMR (400 MHz) spectrum for compound **31** S15

^{1}H NMR (400 MHz) spectrum for compound **32** S16

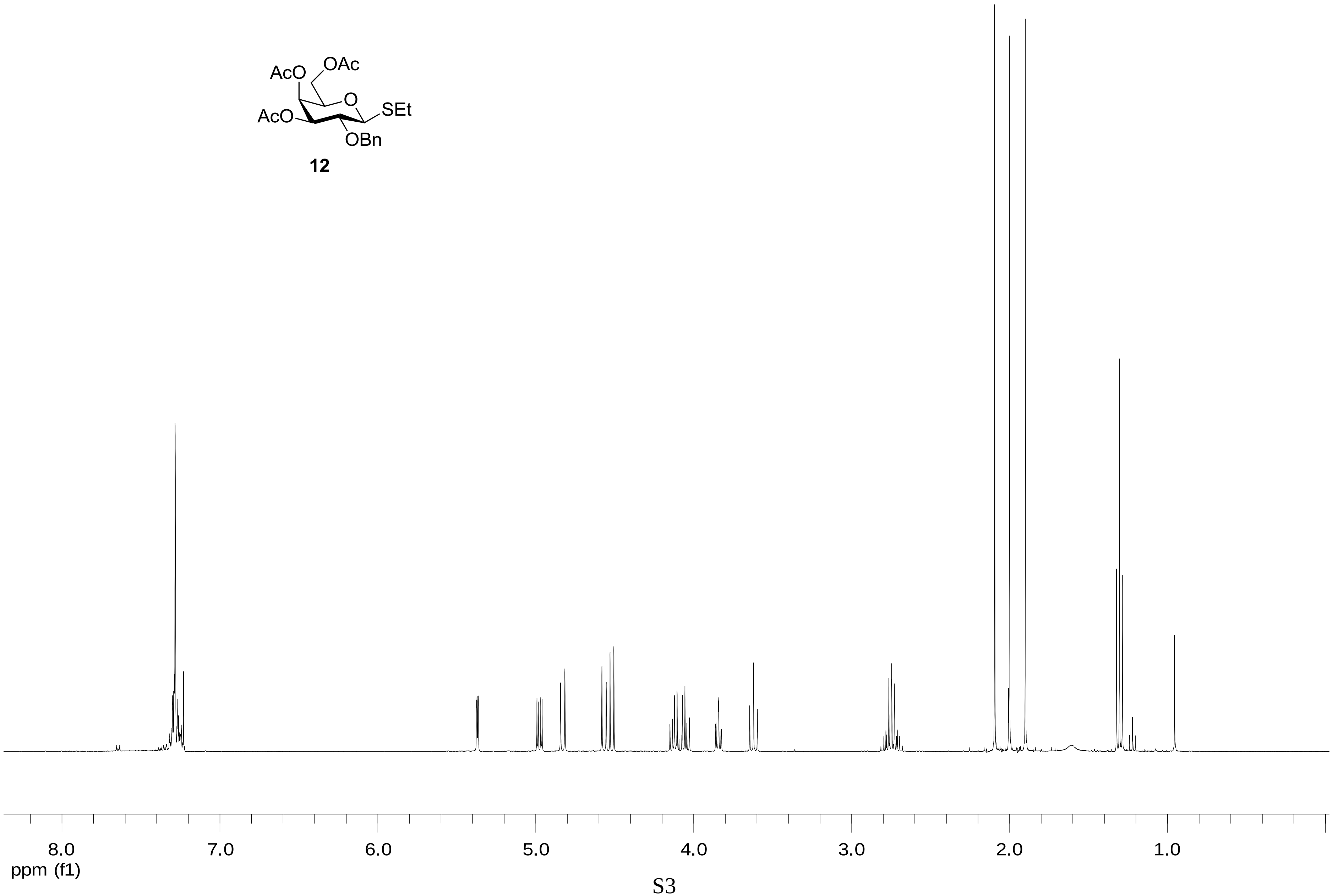

12

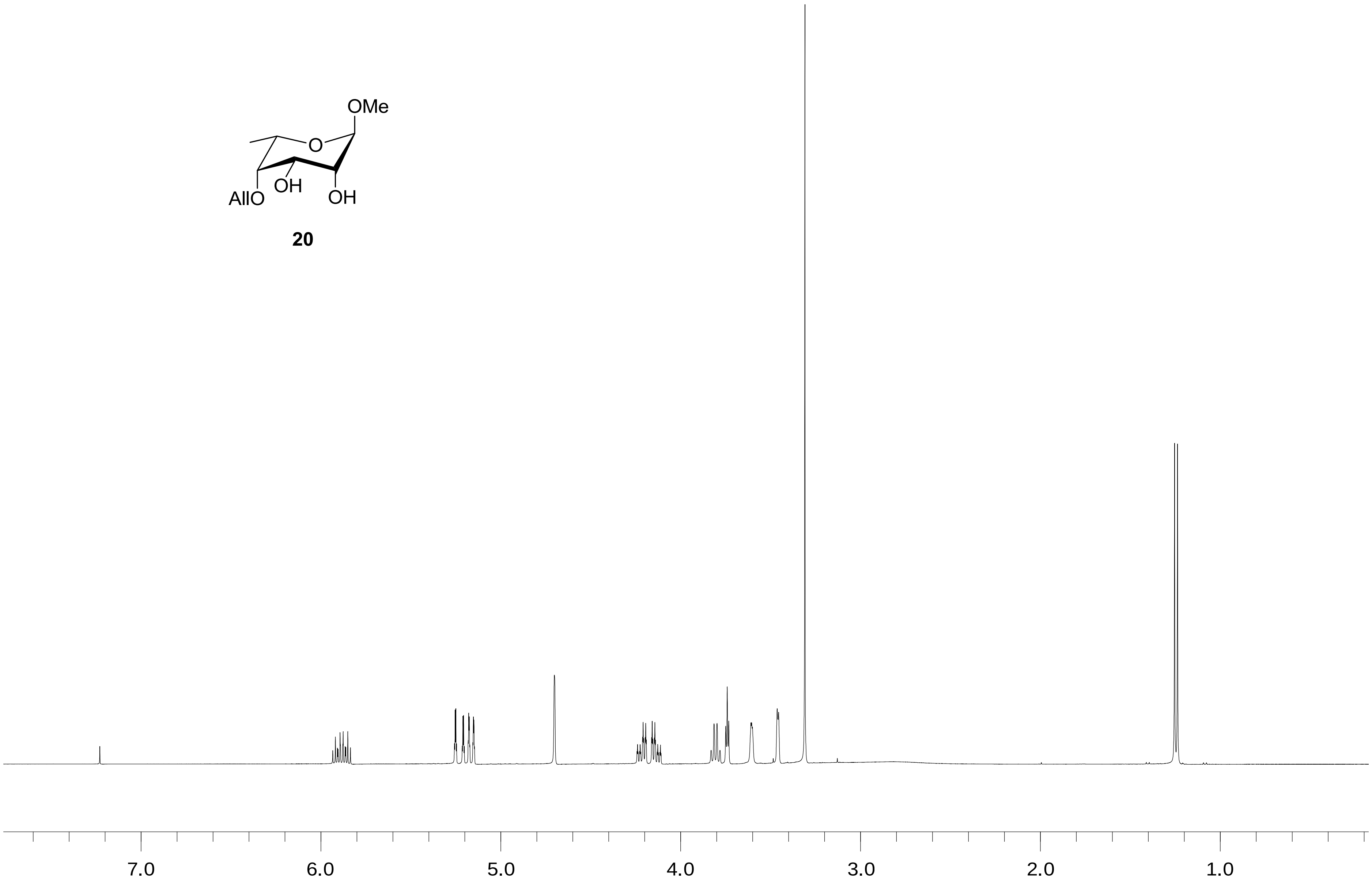
OMe
O
AllO
OH
OH
20
7.0
6.0
5.0
4.0
3.0
2.0
1.0
ppm (t1)

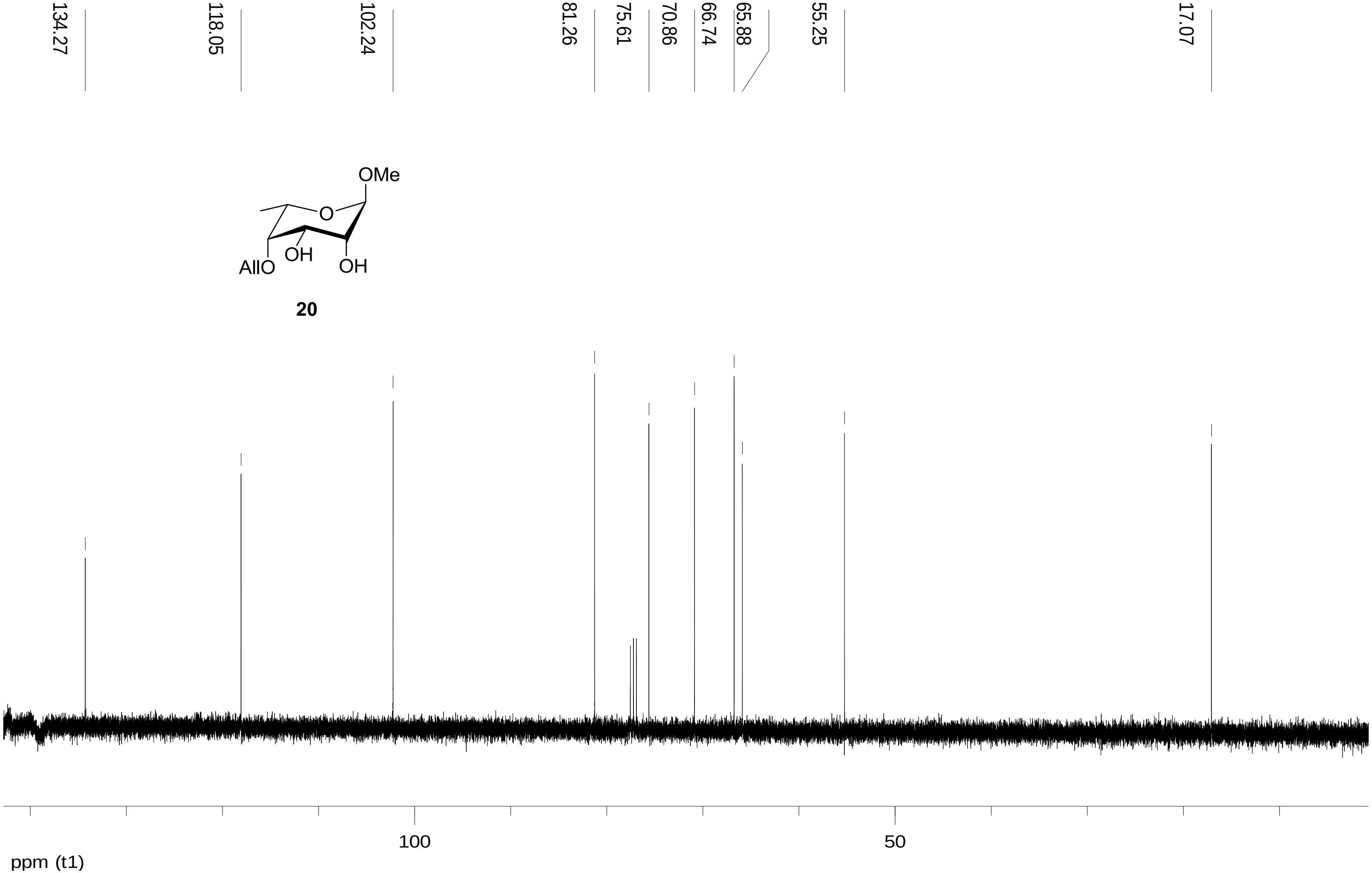
134.27
118.05
102.24
81.26
75.61
70.86
66.74
65.88
55.25
17.07
OMe
O
AllO
OH
OH
20
100
50
ppm (t1)

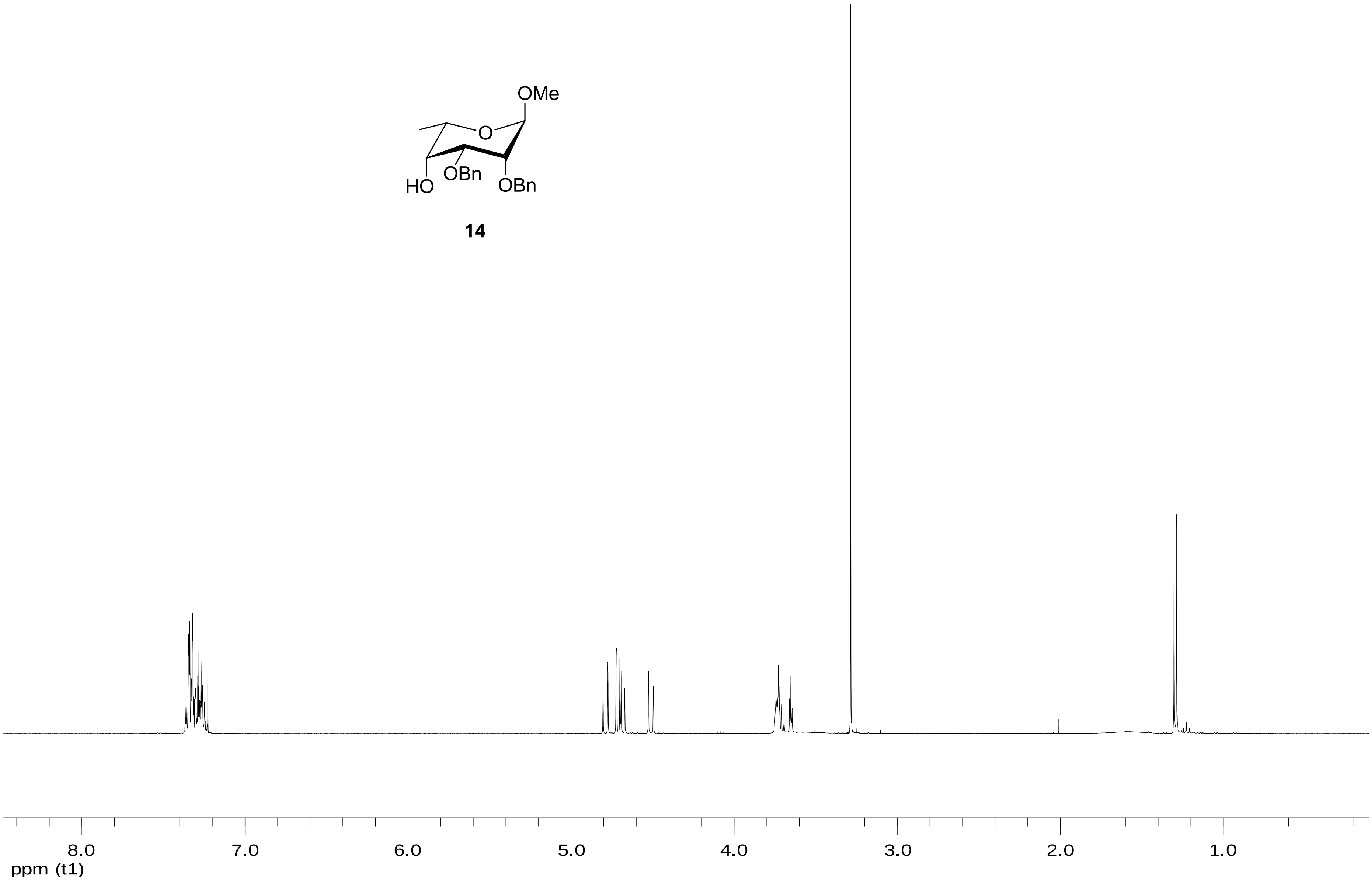
OMe
O
HO
OBn
OBn
14
8.0
7.0
6.0
5.0
4.0
3.0
2.0
1.0
ppm (t1)

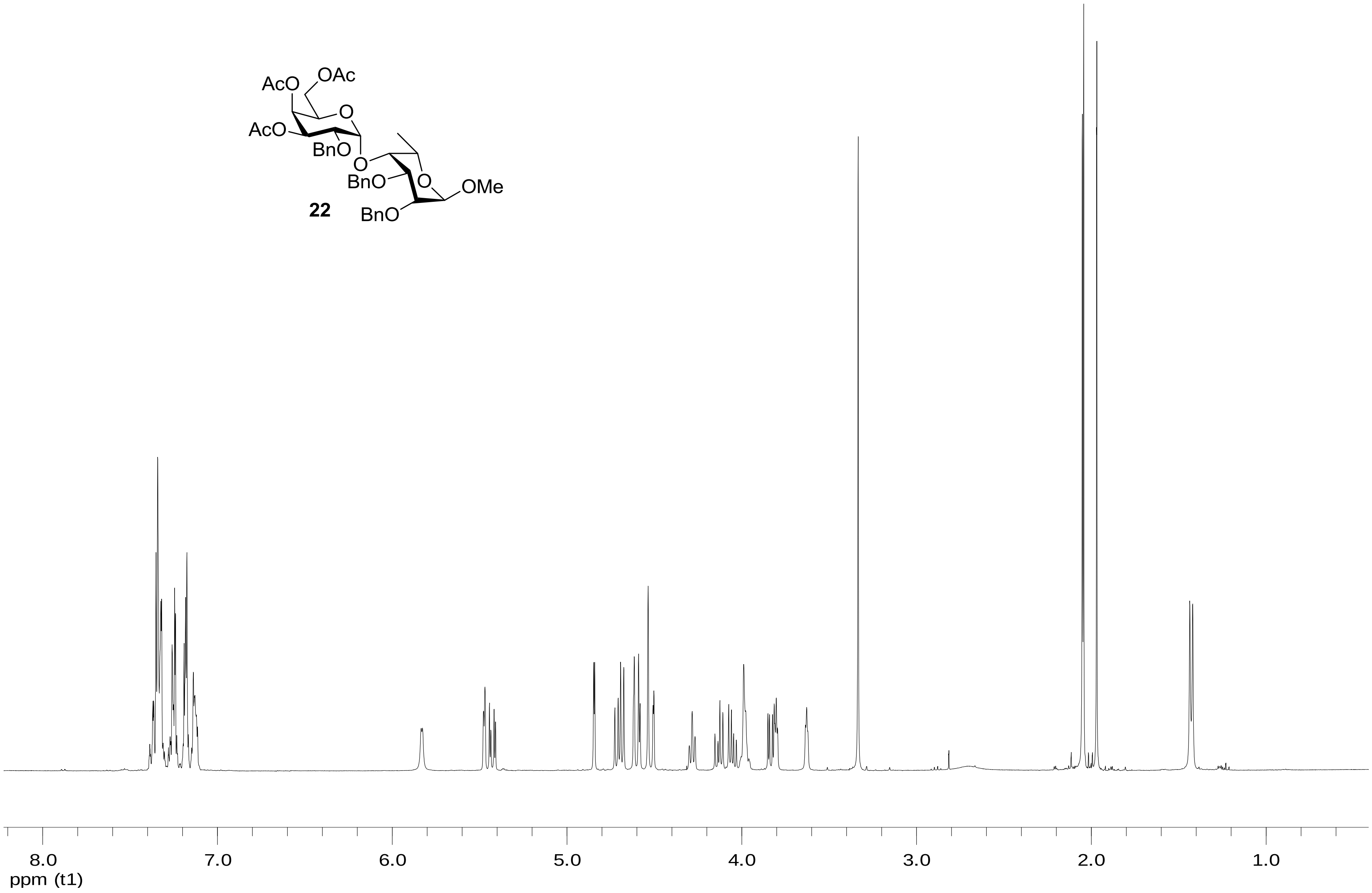
AcO
OAc
O
AcO
BnO
O
BnO
O
OMe
BnO
22
8.0
7.0
6.0
5.0
4.0
3.0
2.0
1.0
ppm (t1)

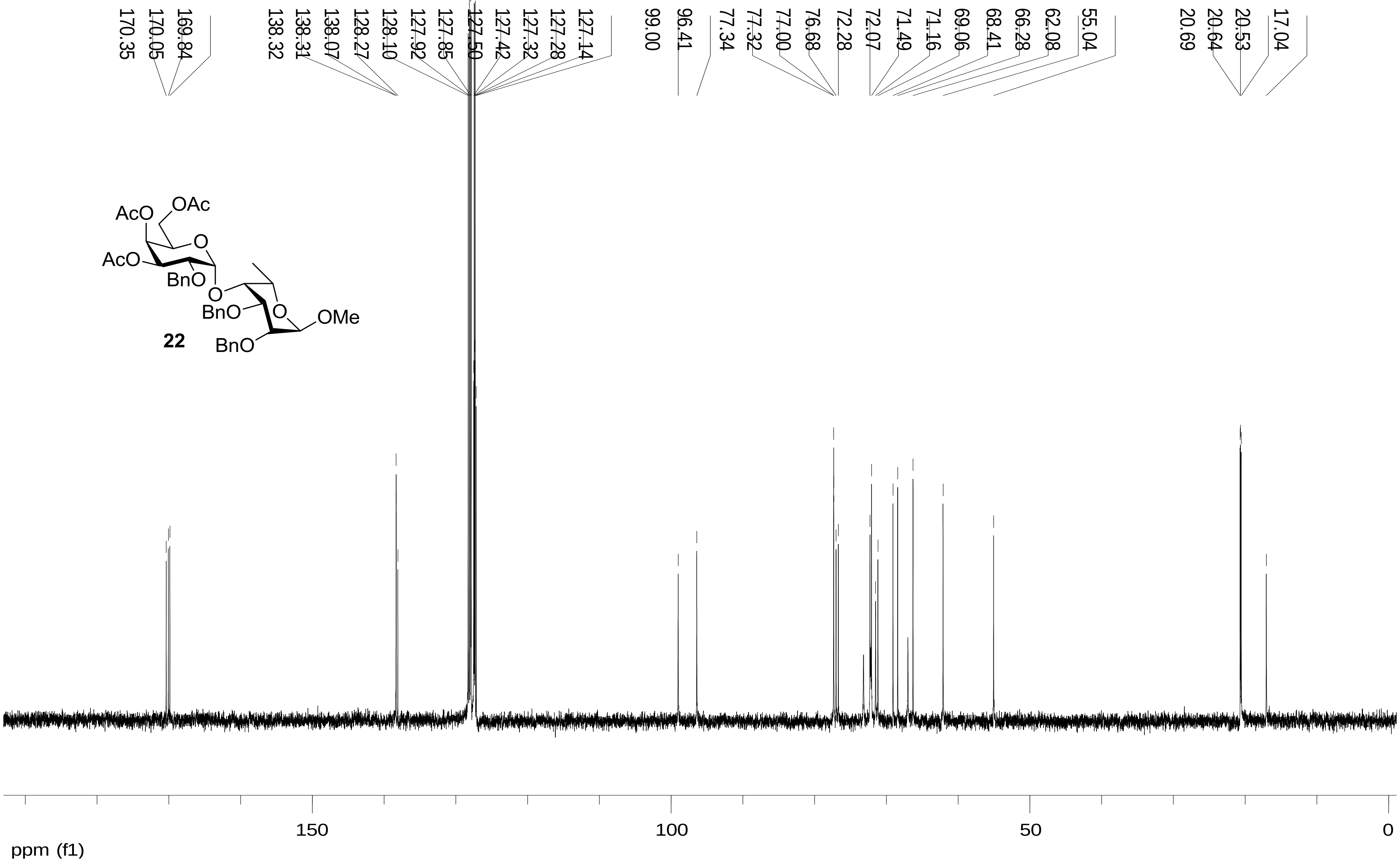
170.35
170.05
169.84
138.32
138.31
138.07
128.27
128.10
127.92
127.85
127.50
127.42
127.32
127.28
127.14
99.00
96.41
77.34
77.32
77.00
76.68
72.28
72.07
71.49
71.16
69.06
68.41
66.28
62.08
55.04
20.69
20.64
20.53
17.04
AcO
OAc
O
AcO
BnO
O
BnO
O
OMe
BnO
22
150
100
50
0
ppm (f1)

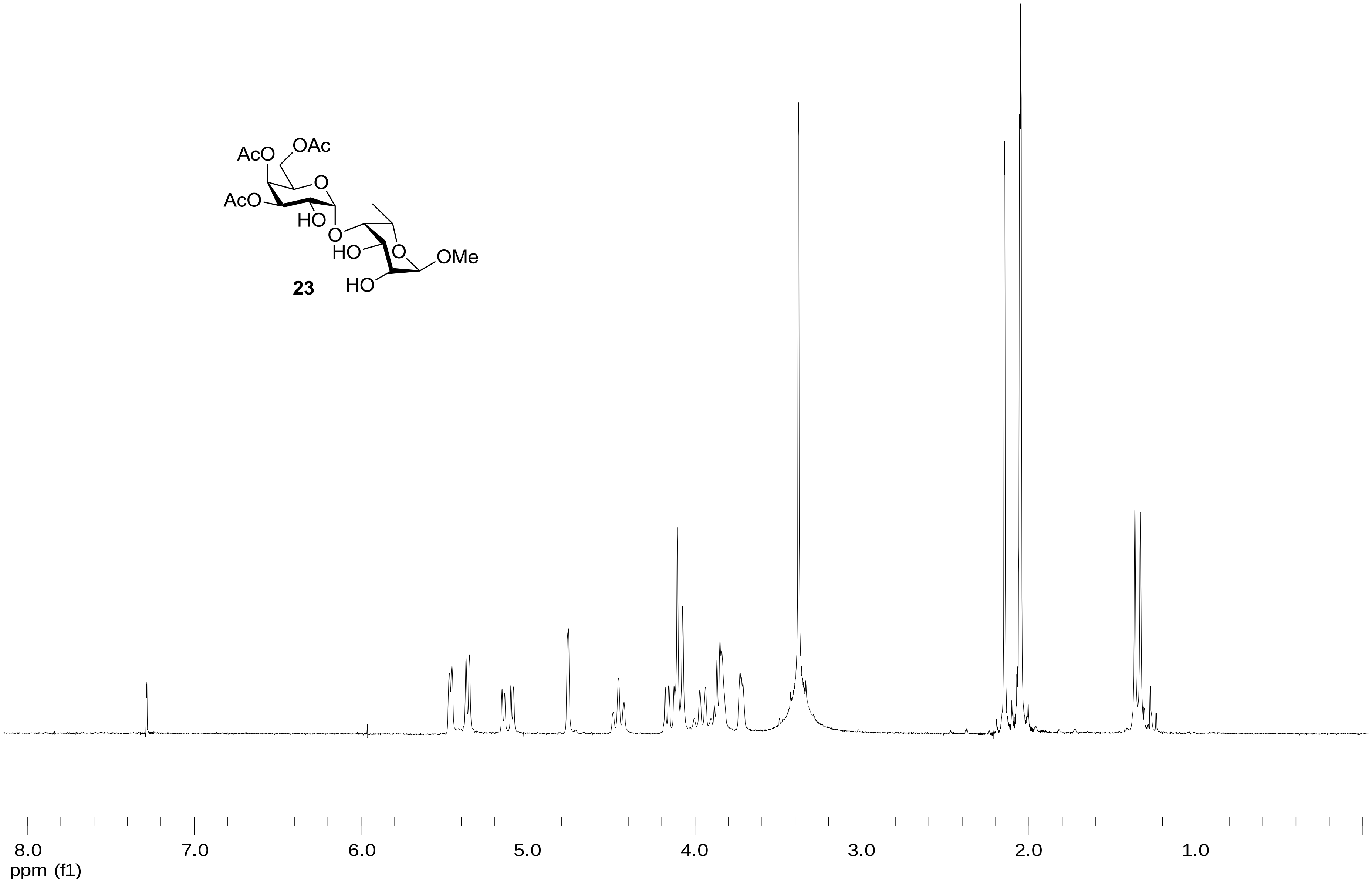
AcO
OAc
AcO
O
HO
O
HO
O
OMe
HO
23
8.0
7.0
6.0
5.0
4.0
3.0
2.0
1.0
ppm (f1)

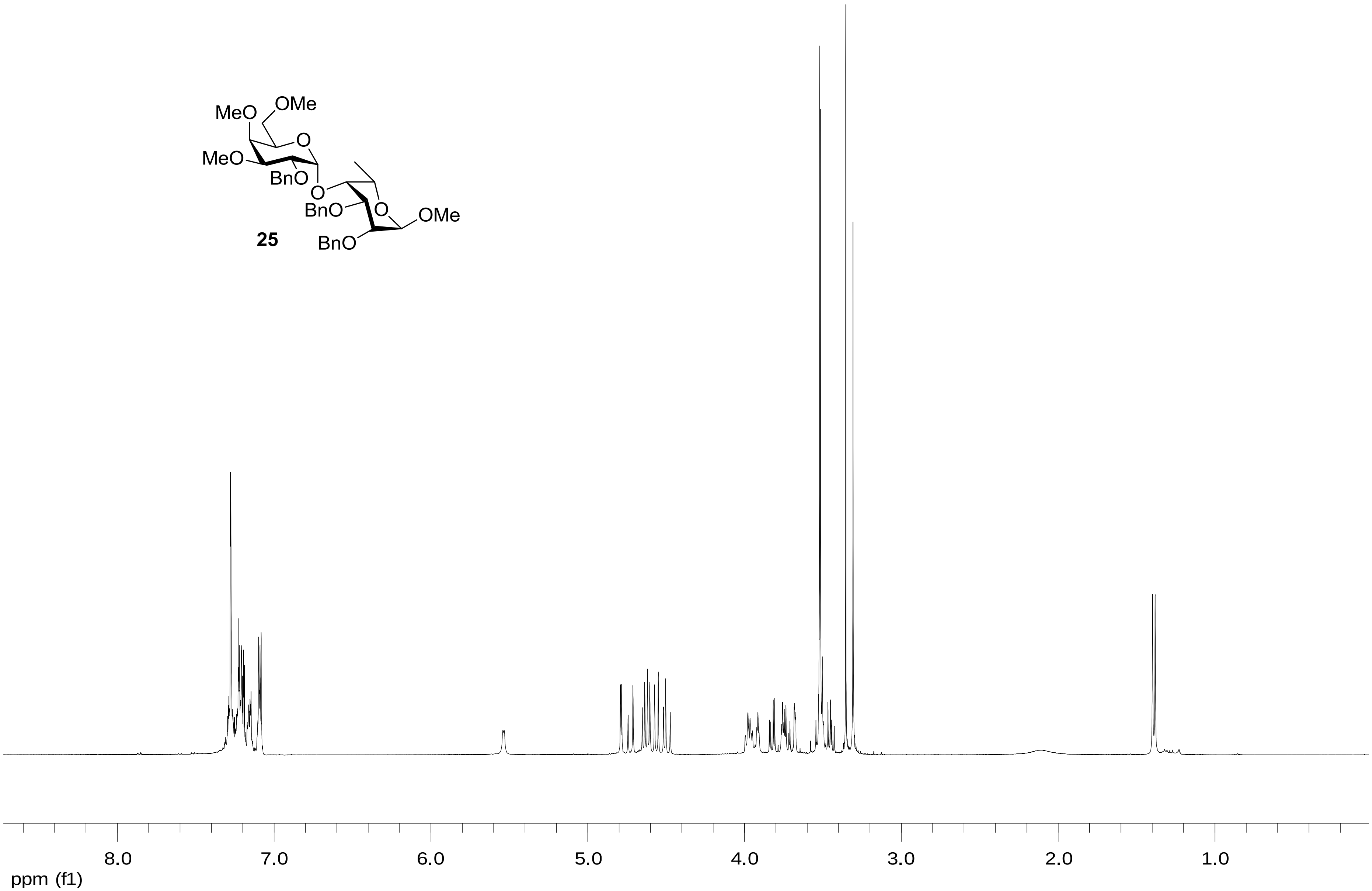

MeO
OMe
O
MeO
BnO
O
BnO
O
OMe
BnO
25
8.0
7.0
6.0
5.0
4.0
3.0
2.0
1.0
ppm (f1)

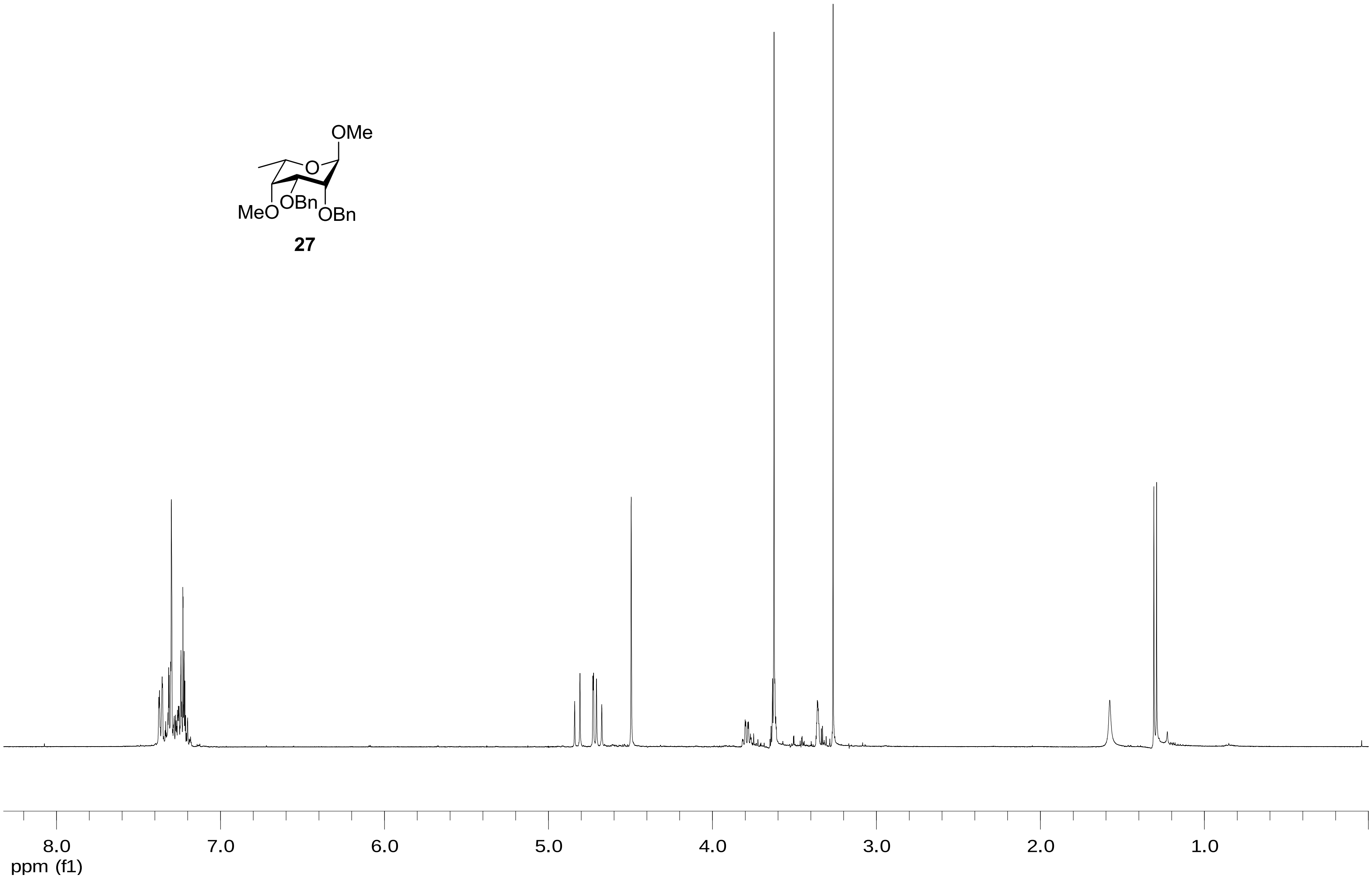

27

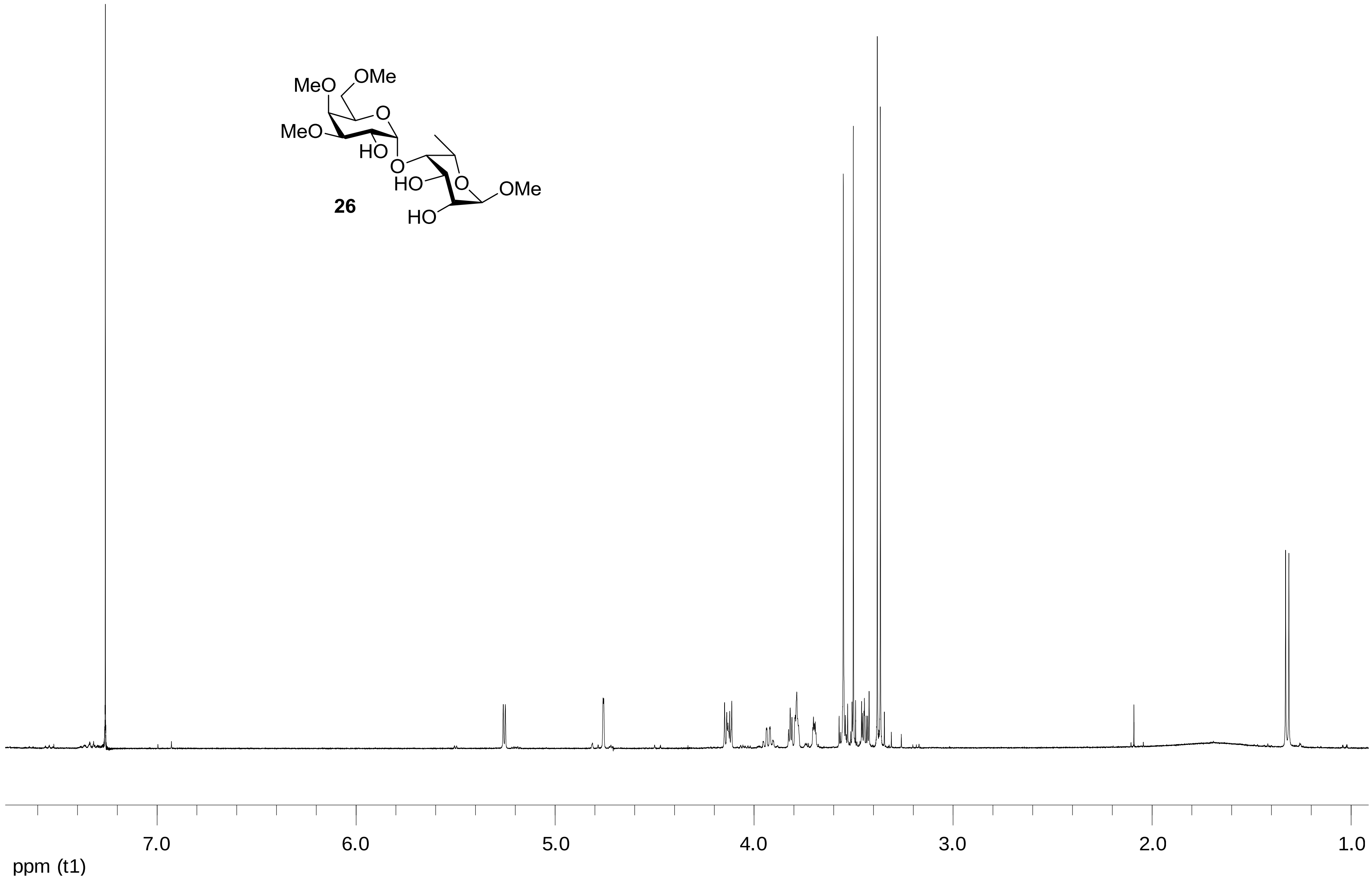
MeO
OMe
O
MeO
HO
O
HO
O
OMe
HO
26
7.0
6.0
5.0
4.0
3.0
2.0
1.0
ppm (t1)

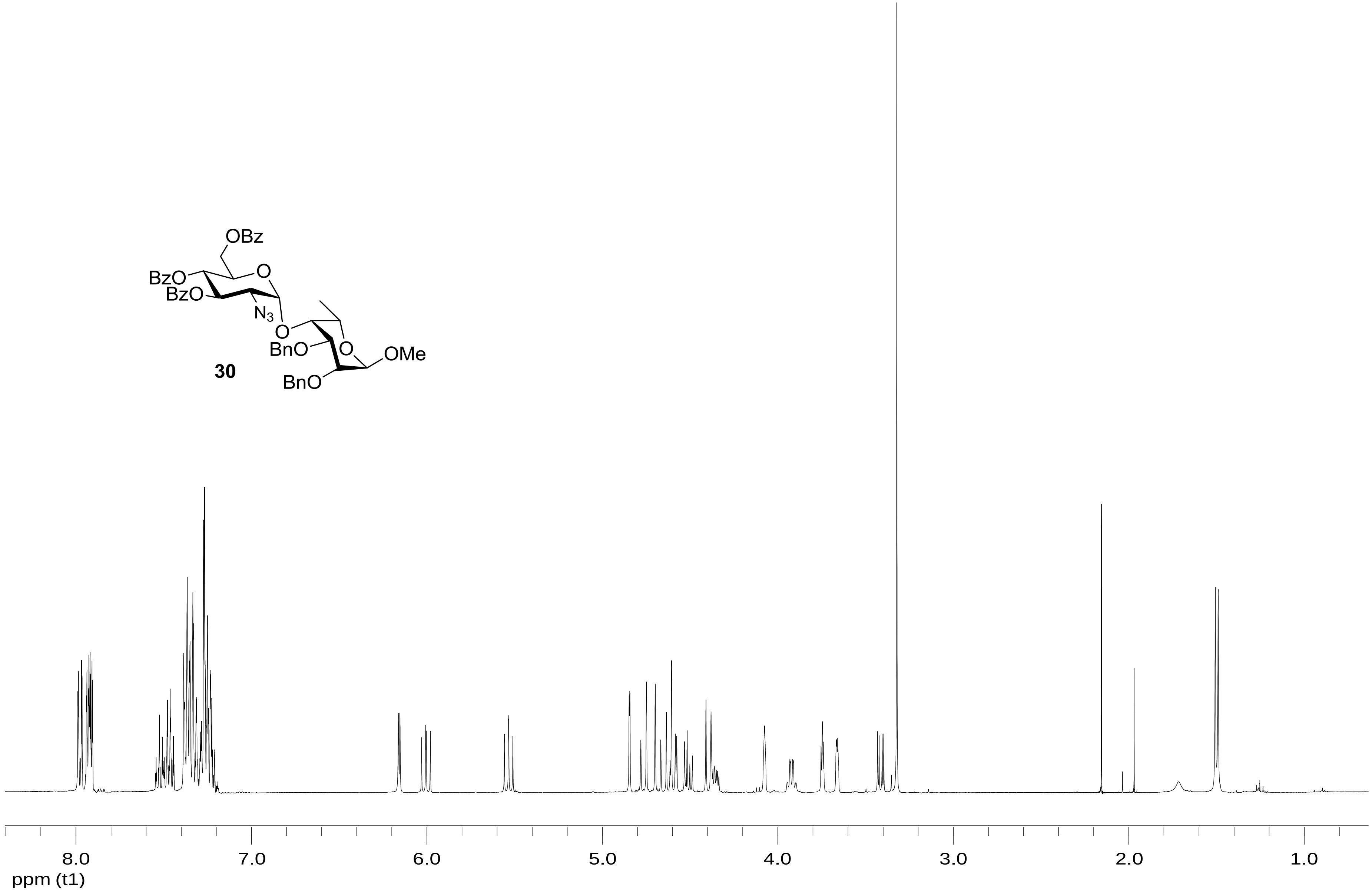
OBz
BzO
BzO
O
N3
O
BnO
O
OMe
BnO
30
8.0
7.0
6.0
5.0
4.0
3.0
2.0
1.0
ppm (t1)

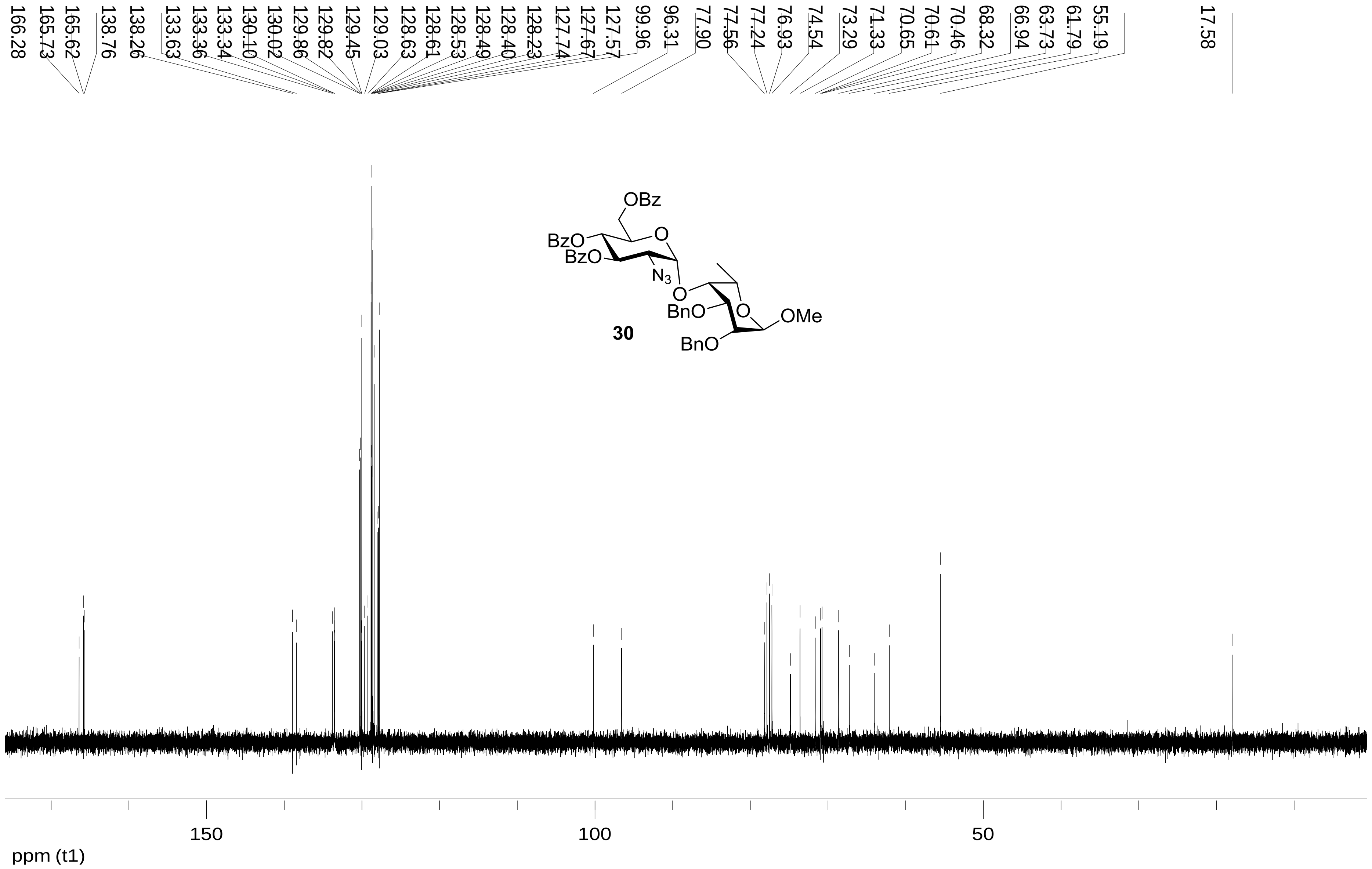
166.28
165.73
165.62
138.76
138.26
133.63
133.36
133.34
130.10
130.02
129.86
129.82
129.45
129.03
128.63
128.61
128.53
128.49
128.40
128.23
127.74
127.67
127.57
99.96
96.31
77.90
77.56
77.24
76.93
74.54
73.29
71.33
70.65
70.61
70.46
68.32
66.94
63.73
61.79
55.19
17.58
OBz
BzO
BzO
O
N_3
O
BnO
O
OMe
BnO
30
150
100
50
ppm (t1)

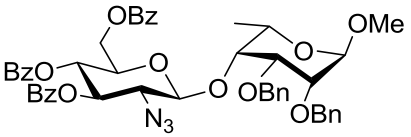

31

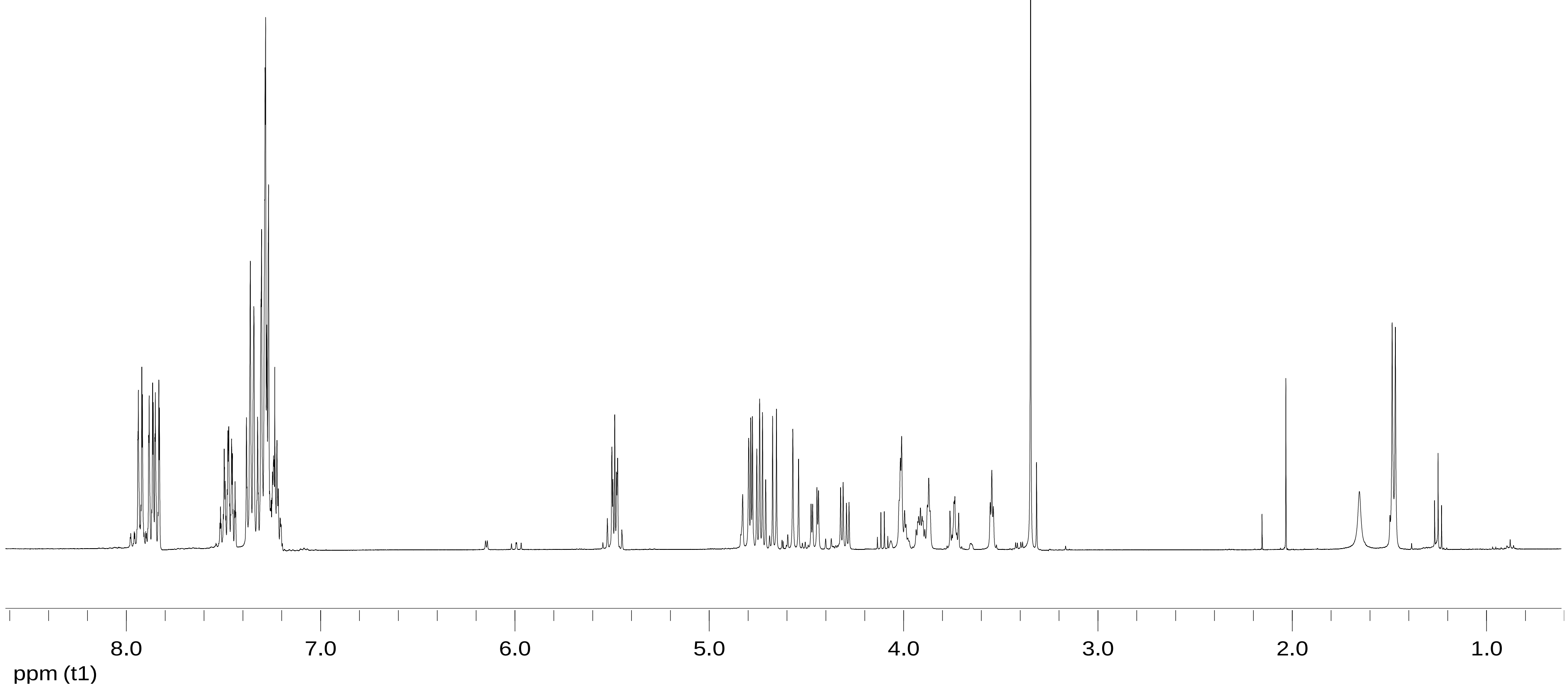

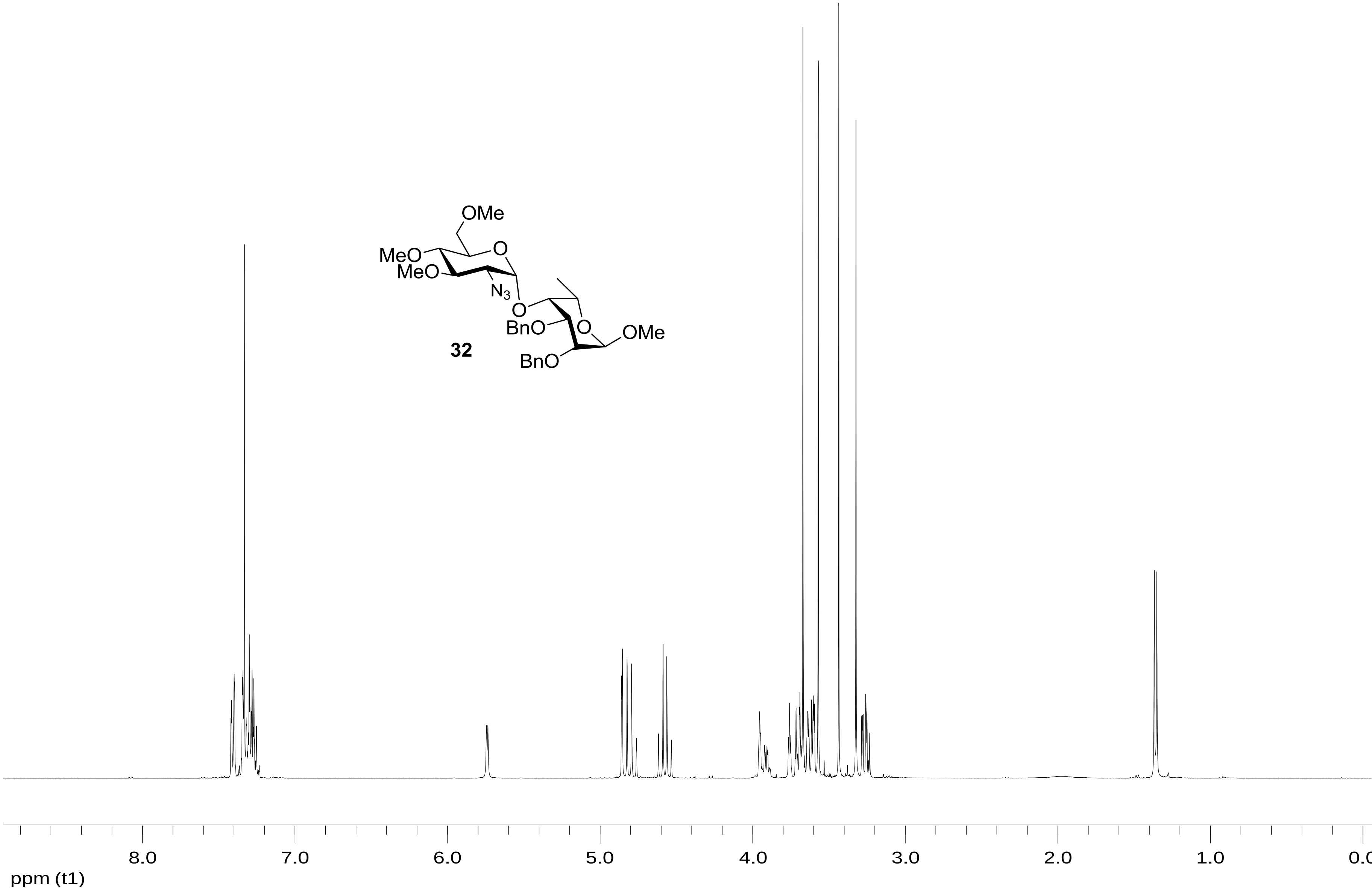
OMe
MeO
MeO
O
N3
O
BnO
O
OMe
BnO
32
8.0
7.0
6.0
5.0
4.0
3.0
2.0
1.0
0.0
ppm (t1)
